# Hidden Gems in the Transcriptome Maps of Competent Streptococci

**DOI:** 10.3389/fmolb.2018.00116

**Published:** 2019-01-04

**Authors:** Roger Junges, Gabriela Salvadori, Tsute Chen, Donald A. Morrison, Fernanda C. Petersen

**Affiliations:** ^1^Faculty of Dentistry, Institute of Oral Biology, University of Oslo, Oslo, Norway; ^2^Department of Microbiology, The Forsyth Institute, Cambridge, MA, United States; ^3^Department of Biological Sciences, College of Liberal Arts and Sciences, University of Illinois at Chicago, Chicago, IL, United States

**Keywords:** pheromone, competence, natural transformation, mutagenesis, oral cavity, CSP, streptococcus

## Abstract

Natural transformation is regarded as an important mechanism in bacteria that allows for adaptation to different environmental stressors by ensuring genome plasticity. Since the discovery of this phenomenon in *Streptococcus pneumoniae*, remarkable progress has been made in the understanding of the molecular mechanisms and pathways coordinating this process. Recently, the advent of high-throughput sequencing allows the posing of questions that address the system at a larger scale but also allow for the creation of high-resolution maps of transcription. Thus, while much is already known about genetic competence in streptococci, recent studies continue to reveal intricate novel regulation pathways and components. In this perspective article, we highlight the use of transcriptional profiling and mapping as a valuable resource in the identification and characterization of “hidden gems” pertinent to the natural transformation system. Such strategies have recently been employed in a variety of different species. In *S. mutans*, for example, genome editing combined with the power of promoter mapping and RNA-Seq allowed for the identification of a link between the ComCDE and the ComRS systems, a ComR positive feedback loop mediated by SigX, and the XrpA peptide, encoded within *sigX*, which inhibits competence. In *S. pneumoniae*, a novel member of the competence regulon termed BriC was found to be directly under control of ComE and to promote biofilm formation and nasopharyngeal colonization but not competence. Together these new technologies enable us to discover new links and to revisit old pathways in the compelling study of natural genetic transformation.

## The Mine of Natural Genetic Transformation in Streptococci

Natural transformation is the only bacterial horizontal gene transfer (HGT) mechanism in which the recipient cell has primary control over the transfer process. This is in contrast with conjugation and transduction, where the transfer relies primarily on genetic elements in the donor cells. Natural transformation allows microbes to better adapt to different stressors by assimilating divergent genetic information to ensure plasticity of their genomes, correct detrimental mutations, and spread important traits, such as resistance to antibiotics (Johnston et al., 2014; Fontaine et al., 2015; Straume et al., 2015). In addition, natural transformation has for decades been a valuable resource in microbiology as means to create mutant strains.

The discovery of natural transformation in bacteria was first reported in 1928 by Frederick Griffith with his studies on *Streptococcus pneumoniae* (Griffith, 1928). Over the ensuing 90 years, analysis of underlying mechanisms by classical genetic tools and monitoring of gene expression by transcriptional gene fusions and Northern blots gradually revealed intricate regulatory mechanisms that coordinate transient abilities to lyse nearby target cells and to take up and integrate genes released by that attack (reviewed by Fontaine et al., 2015; Straume et al., 2015; Shanker and Federle, 2017). Key elements include secreted “quorum sensing” peptide pheromone signals that coordinate competence development among cooperating cells (Håvarstein et al., 1995), and an alternative sigma factor (SigX) that responds to these signals to orchestrate expression of a large regulon of effector genes within cells (Lee and Morrison, 1999). Hybridization microarrays expanded the roster of genes apparently under control of these circuits (Rimini et al., 2000; Peterson et al., 2004; Vickerman et al., 2007), but their low resolution allowed some elements to be missed and others to be misinterpreted.

With the proliferation of high-throughput tools for parallel measurement of gene expression and the higher precision analysis offered by massive RNA sequencing methods, it has recently become possible to refine the understanding of competence regulation by identification of new actors and new linkages between known actors (Khan et al., 2016; Aprianto et al., 2018; Salvadori et al., 2018). Here we highlight several cases among streptococcal competence systems where new elements or new links are revealed or old interpretations corrected.

## Mining the Hidden Gems

The distribution of competence among streptococcal species long remained obscure. After competence gene expression was found to depend on a peptide pheromone (CSP; Håvarstein et al., 1995) and a two-component signal transduction system (TCSTS) receptor for the peptide (ComDE; Håvarstein et al., 1996; Pestova et al., 1996), the restriction of orthologous quorum sensing (QS) circuits to the mitis and anginosus groups of streptococci raised doubts as to functional competence regulons in species of the other groups, among which reports of transformation were largely sporadic at best (Martin et al., 2006).

A completely different class of peptide-mediated QS regulation of competence genes was not discovered until the application of a new tool for high-throughput parallel determination of gene expression. Use of LC-MS quantitation of tryptic digests of hundreds of proteins to analyze gene expression changes in *Streptococcus thermophilus* when peptide import was blocked revealed an actively-regulated competence regulon in this species of the salivarius group, and led to discovery of control of *sigX* expression by an Rgg class receptor/peptide QS circuit mediated by a small peptide pheromone, named ComR/S (Fontaine et al., 2010; Fleuchot et al., 2011). Remarkably, all streptococcal species outside the mitis and anginosus groups regulate their SigX sigma factors through ComR/S circuits (Fontaine et al., 2013). Exceptionally, *S. mutans* engages both classes of QS circuits for competence regulation (Song et al., 2013; Shanker and Federle, 2017). Identification of characteristic consensus cis-acting regulatory site sequences for the three key competence regulators, ComE, SigX, and ComR, the “ComE-box” (aCAnTTcaG-12-aCAgTTgaG; Ween et al., 1999), the “SigX-box” (TACGAATA; Campbell et al., 1998), and the ComR-box (GACA-12-TGTC; Fontaine et al., 2013), facilitates the interpretation of high-resolution transcript maps.

After the discovery of the ComRS cell-to-cell signaling system in the salivarius group (Fontaine et al., 2010), an orthologous circuit was identified in *S. mutans*, where it regulates competence via XIP, a 7-amino-acid peptide pheromone derived from ComS (Mashburn-Warren et al., 2010; Khan et al., 2012). Under alternative conditions, competence in *S. mutans* can be stimulated either by an 18-aa CSP acting through a ComD receptor (Petersen et al., 2006; Hossain and Biswas, 2012) or by XIP, acting through a ComR receptor (Mashburn-Warren et al., 2010; Khan et al., 2012). A link between the two regulatory pathways remained elusive until 2015, when a binding site for SigX was identified upstream of *comE*, where it could link ComRS-regulated SigX to expression of ComED (aka BlpRH) pathway for control of bacteriocin production (Reck et al., 2015; Son et al., 2015; Khan et al., 2016). To uncover this new regulatory link, the authors initially combined the use of dual-reporter strains for assessment of population heterogeneity with RNA-seq data to determine gene expression in regulatory mutants (Reck et al., 2015). Additional studies arrived to the same conclusion through different approaches (Son et al., 2015; Khan et al., 2016).

In *S. mutans*, the presence of an intragenic SigX-box within the ORF SMU.60 came under scrutiny particularly because *comR* is immediately downstream of that gene and early microarray data had indicated an unexplained up-regulation of *comR* during competence (Perry et al., 2009; Okinaga et al., 2010; Dufour et al., 2011; Lemme et al., 2011). High-resolution RNA-Seq mapping of competence-associated transcripts at this locus revealed increased unidirectional RNA synthesis beginning not in the SMU.60-*comR* intergenic region but ~200 bp upstream, within the SMU.60 ORF and closely downstream of a candidate SigX-box. Nested deletions at the 3′ end of SMU.60 allowed the up-regulation of *comR* transcription only when this putative SigX-box remained intact. Three single-base mutations in the consensus SigX-box caused only silent mutations in SMU.60, but significantly decreased the induced *comR* transcription, showing that the presence of the SigX-box was essential for the up-regulation of *comR* at competence (Khan et al., 2017). This gem was discovered by coupling high-resolution RNA-Seq mapping to search for signature boxes upstream of the induced transcript, revealing an unexpected feedback loop for *comR* mediated by its regulatory target, SigX.

A series of studies of competence regulation in *S. mutans* culminated in identification of an open reading frame nested within the *sigX* gene that regulates genetic competence and oxidative stress tolerance in *S. mutans* (Kaspar et al., 2015). Investigating a link between oxidative stress tolerance, (p)ppGpp metabolism, and competence in the *rcrRPQ* operon, the authors observed that an *rcrR* polar (Δ*rcrR*-P) mutant was hypertransformable while a non-polar (Δ*rcrR*-NP) mutant did not transform. The expression levels of the *sigX* gene as a whole were 100-fold higher in both mutants compared to the WT (Seaton et al., 2011); however, the number of reads mapped to the 5′ region of *sigX* in the Δ*rcrR*-NP mutant were much less than in the Δ*rcrR*-P mutant. Conversely, in the central and 3′ regions of *sigX*, the expression pattern was reversed, revealing that a sense transcript located within the 3′ portion of *sigX* was highly expressed in the Δ*rcrR*-NP mutant. The level of full-length *sigX* transcript was actually reduced in the non-polar mutant, despite both transcripts being expressed from the same promoter. This internal transcript is translated from a second reading frame, yielding a 69-aa peptide product, termed *sigX* regulatory peptide A (*xrpA*). SigX production and transformation levels were restored when *xrpA* expression was disrupted by introduction of two silent mutations within *sigX* in the Δ*rcrR*-NP background. Furthermore, the target of XrpA has been traced directly to the binding of the ComR-XIP complex to the *sigX* promoter (Kaspar et al., 2018). The gem in this case was hidden in plain sight as a transcript internal to one of the most studied genetic components of natural transformation: SigX. However, it was revealed only when the power of RNA-seq quantitatively distinguished transcripts representing different regions of *sigX* (Kaspar et al., 2015).

The pneumococcal transcriptome was recently assessed by RNA-Seq in 22 different conditions relevant to infection, including competence for genetic transformation (Aprianto et al., 2018). Upon investigating genes with expression correlating with expression of *comCDE*, the authors identified a candidate that had not been noticed in two previous array-based studies of the competence regulons. Expression levels of this gene were strongly correlated with expression of *comCDE* across infection-relevant conditions, and directly repeated *comE* boxes in its promoter region indicated direct regulation by ComE. In addition, searches for double glycine leaders at the N-termini of pneumococcal proteins had identified 25 sequence-clusters within a predicted “secretome” including this same product (Cuevas et al., 2017). Interestingly, deletion of the candidate gene had no effect on transformation (Aprianto et al., 2018), but rather affected biofilm formation and nasopharyngeal colonization (Aggarwal et al., 2018; Aprianto et al., 2018); thus, it was termed *B*iofilm-*r*egulating peptide *i*nduced by *c*ompetence (BriC) and added to the list of genes under regulation in *S. pneumoniae* during competence (Cuevas et al., 2017; Aggarwal et al., 2018; Aprianto et al., 2018). This hidden gem was revealed by combining the power of large numbers of WGS for individual streptococcal species to identify conserved structures with the high-throughput of RNA-Seq to reveal correlated patterns of expression.

## Avoiding the Fool's Gold

The early data on the transcriptional response during genetic competence were produced using strategies of microarray construction and analysis that were known to have technical limitations, including a lack of strand specificity, the use of probes restricted to annotated ORFs, the use of probes with low spatial resolution, and the use of cDNA preparation techniques that can result in false positives. In contrast, the current use of RNA-sequencing for transcriptome analyses enables improved genome coverage, a higher spatial resolution, strand specificity, analysis of intergenic or unannotated genomic regions, and provides improved mapping of candidate promoter sites (Vivancos et al., 2010; Mills et al., 2013; Zhao et al., 2015). During searches for hidden gems using early transcriptome approaches, it is not rare to encounter fool's gold. For instance, the gene SMU63c was reported as being upregulated during competence (Reck et al., 2015; Khan et al., 2016; Wenderska et al., 2017). However, when re-examined using directional RNA-seq (Khan et al., 2017), the apparent competence-associated transcription of SMU63.c was restricted to the anti-sense direction. In fact, transcription of SMU63c in the sense direction was actually down-regulated 35-fold.

Other false positives, arising through differential expression of genes located downstream of SigX-boxes but in the antisense direction (Khan et al., 2016), highlight the possibility of read-through transcripts without biological significance. Such findings result both from lack of strand-specific information, as detailed in the case above, and from pervasive transcription due to inefficient or missing transcription termination signals. Regarding the competence response particularly, pervasive transcription may be responsible for the upregulation of many so-called “non-core” genes of the competence response in various species (Khan et al., 2016), which comprise mostly genes with yet unknown functions. Although it represents a widespread phenomenon in bacteria (Wade and Grainger, 2014), we cannot rule out that some instances of pervasive transcription might also contribute to the optimization of the competent state. On the other hand, the lack of inter-species conservation of competence-specific pervasive transcripts in bacteria suggests that they lack such a functional role (Raghavan et al., 2012). In the case of competence operons, there may simply be little selective pressure favoring rigorous termination of transcripts that are perhaps made rarely and for a short time.

## Finding the right conditions

Finding the right environmental and experimental conditions for competence has been the key to successful research throughout the history of discoveries on transformation, starting with numerous efforts to reproduce *in vitro* the observations by Griffith of transformation in mice (Griffith, 1928). Growth in the presence of anti-serum was one of the factors that seemed to do the trick (Bracco et al., 1957). However, higher and reproducible levels of competence were only achieved later on, with the discovery of the competence signaling peptide in streptococci of the mitis, anginosus, and mutans groups, and the possibility to use it in a synthetic form (Håvarstein et al., 1995; Li et al., 2001; Petersen et al., 2004).

A remarkable example of combining the identification of optimal growth conditions for competence with proteomics and transcriptomics to reveal a hidden gem came from studies with streptococci of the salivarius group. In this group, the use of a chemically defined medium without peptides, combined with proteomic (Gardan et al., 2009) and transcriptomic (Fontaine et al., 2010) approaches, revealed a new class of quorum sensing signaling that controls competence. The sequence for a Rgg regulator was located close to bacteriocin genes induced during competence, and it was followed by a putative pheromone gene. This was found to encode a competence signaling peptide recognized by the Rgg and internalized by an oligopeptide permease (Opp; Gardan et al., 2009; Fontaine et al., 2010, 2013). This discovery paved the way for the identification of similar systems in the pyogenic, bovis, suis, and mutans groups of streptococci (Mashburn-Warren et al., 2010, 2012; Fleuchot et al., 2011; Khan et al., 2012; Fontaine et al., 2013; Gardan et al., 2013; Morrison et al., 2013; Zaccaria et al., 2014). The reason why the function of this quorum sensing system was only apparent in peptide-free medium is not yet entirely clear, but peptides in the medium may compete with internalization of the competence signal by the oligopeptide permease.

Still, transformable strains and species can often show high differences in transformation levels even with the use of synthetic competence pheromones. This is illustrated by *S. pneumoniae* and *S. mitis*, which can show high levels of transformation efficiency in the laboratory (Hotchkiss, 1958; Ephrussi-Taylor et al., 1965; Lacks, 1966; Salvadori et al., 2016) in comparison to *S. pyogenes*, which rarely transforms despite seemingly possessing all the necessary genetic apparatus (Mashburn-Warren et al., 2012; Marks et al., 2014). Clues to these enigmas can most likely be found by studying the environmental cues surrounding each species. Indeed, a number of experimental conditions, e.g., temperature, pH, nutrient availability, are already known to be able to affect competence for natural transformation (Johnsborg and Håvarstein, 2009; Guo et al., 2014; Moreno-Gamez et al., 2017), and there are likely many more to be discovered. By mastering the use of powerful resources such as transcriptome mapping, we increase our chances to generate relevant and accurate data about these systems, which are thought to play an important role in bacterial adaptation and evolution.

## Gems Awaiting Discovery

In the “mining” process to find real gems, important additional clues have been obtained by obtaining transcriptome maps that reflect different stages during competence development (Peterson et al., 2004; Vickerman et al., 2007; Lemme et al., 2011; Khan et al., 2016). Maps derived from samples with prolonged exposure to pheromone show that the expression of hundreds of genes can be affected in addition to the early and late classes of competence genes, but these are not conserved across the genus and do not seem to include any genes essential for transformation. On the other hand, reducing as much as possible the time point of evaluation following treatment with competence pheromones to obtain snapshots that reflect the sequential events following competence has been particularly revealing. This is illustrated by the fact that all genes identified as essential for transformation that are induced by the competence pheromones are either early or late genes. Comparison of transcriptome maps across streptococcal species using a *S. mutans* detailed map to filter away genes that apparently belong to “the fool's gold” has indeed led to the identification of a core set of 27 pan-streptococcal genes regulated by SigX two thirds of which have a defined function in transformation (Khan et al., 2016). The functions of the remaining third of the core regulon are not yet defined, but due to their conserved presence as part of the SigX regulon across species, these have a high potential to represent gems awaiting the right experimental conditions to reveal their roles.

## Concluding Remarks and Future Perspectives

**Figure 1:**
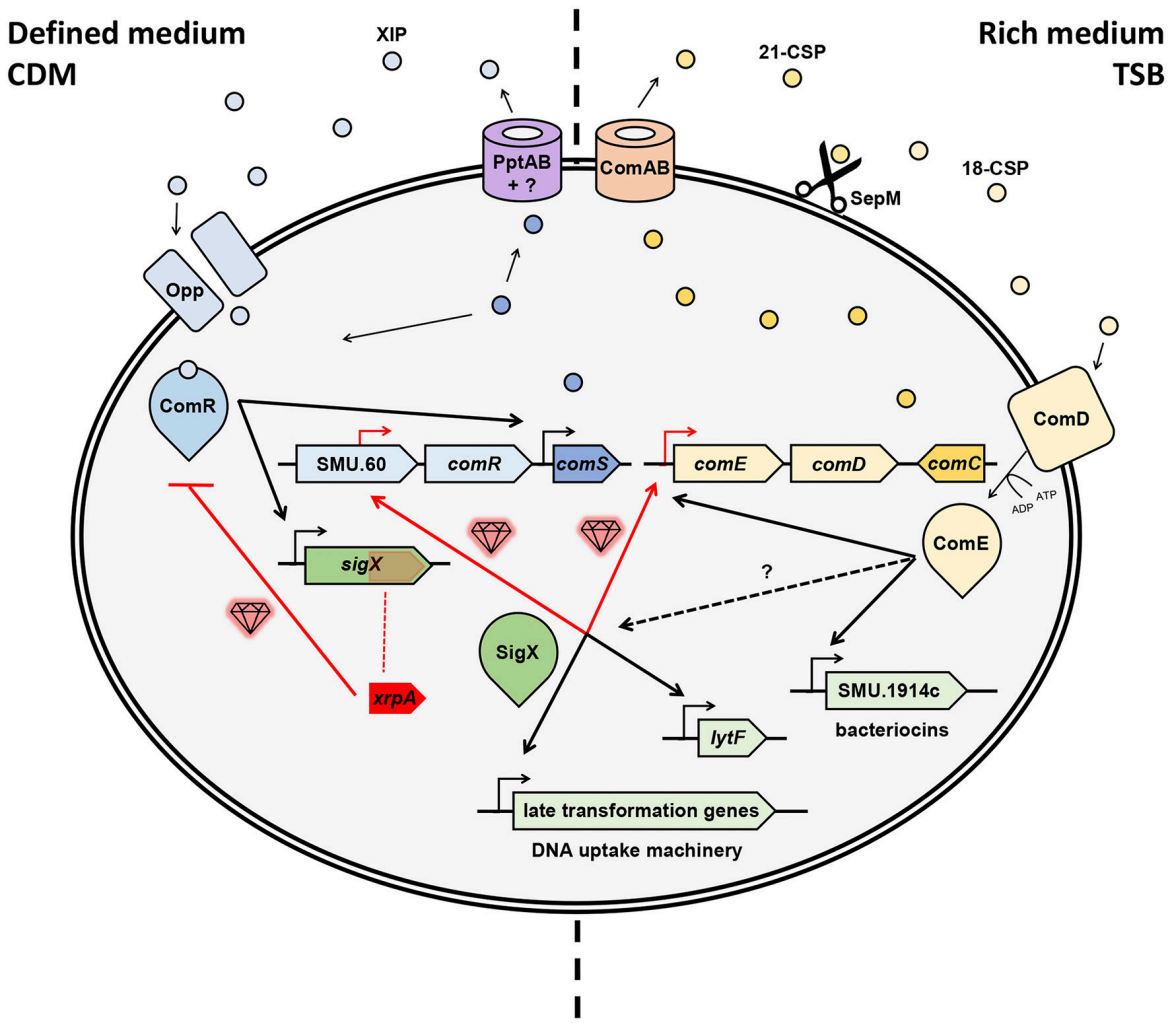
Hidden gems discovered in the regulation of natural genetic transformation in *S. mutans*. Competence can be triggered in chemically defined medium (CDM) by XIP or in rich medium (e.g., TSB) by CSP (aka MIP). Gems illustrated in red indicate novel pathways or elements. In CDM, ComS is exported by PptAB and another unknown element (Chang and Federle, 2016), after which the mature XIP is internalized the via Opp (Gardan et al., 2009), then binds to ComR to activates *sigX* and a *comS* positive feedback loop (Mashburn-Warren et al., 2010; Khan et al., 2012; Fontaine et al., 2013). A “hidden gem” was revealed here as XrpA, an internal peptide to sigX that hinders competence by inhibiting the ComR-XIP complex to bind the promoter region of *sigX* (Kaspar et al., 2015, 2018). Following, SigX, the master regulator of competence, activates transcription of late transformation genes involved in the DNA uptake machinery and also other genes such as lytF (Dufour and Levesque, 2013; Fontaine et al., 2013). Two new gems were found here as SigX recognizes SigX boxes upstream of ComED (Reck et al., 2015; aka BlpRH) and in the central part of SMU.60, upstream of ComR (Khan et al., 2016), and thus proximally regulates *comED* and *comR*. Recently, new evidence indicates that ComS can play a role as both an intercellular and an intracellular signal (Underhill et al., 2018). In rich medium, ComC (aka MIP) is exported by ComAB (aka NlmTE; Hui and Morrison, 1991; Hui et al., 1995; Hale et al., 2005) into 21-CSP, which is then cleaved by SepM (Hossain and Biswas, 2012) into its most active form 18-CSP (Petersen et al., 2006). Once it reaches the threshold, the mature pheromone activates ComD, a histidine kinase (HK) transmembrane receptor, which phosphorylates and, thus, activates the response regulator ComE (similarly as in *S. pneumonia*; Håvarstein et al., 1996; Pestova et al., 1996). Such activation up-regulates the early genes *comABCDE*, creating a positive feedback loop, and activates a variety of bacteriocin genes (Kreth et al., 2007). SigX can also be induced in this pathway through unknown mechanisms.

The contribution of these new “gems” to our understanding of natural genetic transformation in streptococci comes in different shapes and forms. Thus, the findings can highlight a novel ORF or gene that is found embedded in the competence cascade of activation or a novel pathway in the wiring of the competence system. Regardless, these insights together provide the possibility of a more accurate assemblage of the natural genetic transformation puzzle. In the case of *S. mutans*, these regulatory components and pathways recently revealed by high-resolution transcriptional profiling contributed significantly to our appreciation of how the system is wired (Figure 1).

Building a more comprehensive overview of natural genetic transformation and its fine-tuning mechanisms in bacteria is highly warranted considering the relevance of this system in adaptation to different environments and stress response (reviewed by Johnston et al., 2014; Blokesch, 2016). An understanding of the conditions that facilitate or interrupt the development of transformation can aid to the overall comprehension of its role and evolution, in light of the tight evolutionary links between natural transformation and other adaptive responses seen in bacteria, such as the clustered regularly interspaced short palindromic repeats (CRISPRs; Jorth and Whiteley, 2012). We also gain a better insight how the process differs across species that are often inhabiting a similar (or even the same) niche, as it is the case with streptococci, and how this can play a role in their relationship with the host. This is of special relevance in light of the spread of virulence traits and antibiotic resistance demonstrated by a variety of emergent disease isolates that carry new segments of DNA that were likely acquired by HGT (Hiller et al., 2010; Frank et al., 2011; Gottig et al., 2015; Juhas, 2015; Cleary et al., 2016; Brynildsrud et al., 2018). More current than ever, the study of natural genetic transformation and other forms of HGT is a top priority in the global scenario.

## Author Contributions

RJ, GS, TC, DM, and FP conceptualized the work, drafted, and critically revised the article. All authors approved the final version to be published.

### Conflict of Interest Statement

The authors declare that the research was conducted in the absence of any commercial or financial relationships that could be construed as a potential conflict of interest.
